# Understanding the Models of Community Hospital rehabilitation Activity (MoCHA): a mixed-methods study

**DOI:** 10.1136/bmjopen-2015-010483

**Published:** 2017-02-27

**Authors:** John Gladman, John Buckell, John Young, Andrew Smith, Clare Hulme, Satti Saggu, Mary Godfrey, Pam Enderby, Elizabeth Teale, Roberto Longo, Brenda Gannon, Claire Holditch, Heather Eardley, Helen Tucker

**Affiliations:** 1 Division of Rehabilitation and Ageing, School of Medicine, University of Nottingham, Nottingham, UK; 2 School of Public Health, Yale University, New Haven, USA; 3 Academic Unit of Elderly Care and Rehabilitation, University of Leeds, Leeds, UK; 4 Academic Unit of Health Economics, University of Leeds, Leeds, UK; 5 ScHARR, University of Sheffield, Sheffield, UK; 6 NHS Benchmarking Network, Manchester, UK; 7 The Patients Association, London, UK; 8 The Community Hospitals Association, Ilminster, UK

**Keywords:** community hospitals, mixed research methods

## Abstract

**Introduction:**

To understand the variation in performance between community hospitals, our objectives are: to measure the relative performance (cost efficiency) of rehabilitation services in community hospitals; to identify the characteristics of community hospital rehabilitation that optimise performance; to investigate the current impact of community hospital inpatient rehabilitation for older people on secondary care and the potential impact if community hospital rehabilitation was optimised to best practice nationally; to examine the relationship between the configuration of intermediate care and secondary care bed use; and to develop toolkits for commissioners and community hospital providers to optimise performance.

**Methods and analysis:**

4 linked studies will be performed. Study 1: cost efficiency modelling will apply econometric techniques to data sets from the National Health Service (NHS) Benchmarking Network surveys of community hospital and intermediate care. This will identify community hospitals' performance and estimate the gap between high and low performers. Analyses will determine the potential impact if the performance of all community hospitals nationally was optimised to best performance, and examine the association between community hospital configuration and secondary care bed use. Study 2: a national community hospital survey gathering detailed cost data and efficiency variables will be performed. Study 3: in-depth case studies of 3 community hospitals, 2 high and 1 low performing, will be undertaken. Case studies will gather routine hospital and local health economy data. Ward culture will be surveyed. Content and delivery of treatment will be observed. Patients and staff will be interviewed. Study 4: co-designed web-based quality improvement toolkits for commissioners and providers will be developed, including indicators of performance and the gap between local and best community hospitals performance.

**Ethics and dissemination:**

Publications will be in peer-reviewed journals, reports will be distributed through stakeholder organisations. Ethical approval was obtained from the Bradford Research Ethics Committee (reference: 15/YH/0062).

**Strengths and limitations of this study**

- This mixed-methods study will use economic analyses of large, existing, up-to-date databases together with new empirical observational data to provide evidence of value to commissioners and providers about how to optimise the performance in community hospitals which would not be possible using other methods.
- The nature of the economic findings on relative performance will be limited by the size and content of the existing databases which are to be analysed.
- The findings may not generalise beyond the UK setting.

## Introduction

There are 10 million people in the UK who are over 65 years old. Between 2010 and 2035, the proportion of the population aged 65 and over is expected to rise from around 16% to around 23%; and the proportion of the population aged 85 and over from around 2% to around 5%.^1^ A growing older population will be accompanied by an increasing impact on health and social care services. Decision-makers across the National Health Service (NHS) and local government are attempting to reconfigure services in response.

Rehabilitation lies at the heart of best practice for older people. Rising emergency admissions and reductions in acute hospital beds, leading to shorter lengths of stay, limits the scope for rehabilitation in general hospitals.^2^ Intermediate care provides short-term, community-based support and rehabilitation, delivered using a range of service models including community hospitals, care homes and home based. The UK National Audit of Intermediate Care (NAIC)^3^ has implied an underprovision of intermediate care services and insufficient whole system impact. There is uncertainty both about what type of intermediate care works and what configuration of intermediate care services offers a comprehensive repertoire of provision.

Community hospitals are part of this uncertainty about intermediate care. Community hospitals are local hospitals providing a range of healthcare facilities and resources that usually does not include emergency or acute medical inpatient care, intensive care or major surgery, but very commonly includes inpatient rehabilitation for older people. Community hospitals are long established in the UK and internationally.^4–7^ High-quality evidence supports community hospitals as effective bed-based rehabilitation services for older people when compared with general hospitals.^8–10^ The care experience reported by patients in community hospital wards is favoured over that provided in general hospital wards.^8^ As there are nearly 300 community hospitals in the UK,^11^ it is important that community hospitals nationally are organised and configured to deliver these superior outcomes.

Three key findings are apparent from two national surveys, the NHS Benchmarking Network (NHSBN) Community Hospitals Project and the NAIC which between them provide information on 180 community hospitals, approximately two-thirds of UK community hospitals. First, these studies confirm that a core function of the contemporary community hospital is rehabilitation, largely for older people: 97% of community hospitals provide rehabilitation. Second, community hospital wards are extremely variable. Examples of variability include: bed provision per 100 000 weighted population (range <10 to 70); clinical leadership (50% nurse led; 50% consultant led); average length of stay (11–58 days); cost per admission (£3700–£17 500) and cost per day (£140–£450). Third, there is potential for improvement. If a community hospital with 20 beds and 90% occupancy with a length of stay at the 75% quartile (31 days) improved to the 25% quartile (21 days), it would be able to treat ∼100 more patients per year—a 48% increase. Alternatively, if a 20-bed community hospital with a cost per occupied bed day at the 75% quartile (£200/day) improved to the 25% quartile (£110/day) the annual savings would be ∼£650 000.

The reasons behind the variations, and therefore the steps needed to improve, are speculative as no detailed study has been designed and conducted systematically to investigate this issue. There is also a paucity of information available to service planners about the staffing levels, comparative outcomes and efficiencies of community hospitals in relation to alternative forms of community rehabilitation services. The Models of Community Hospital Activity (MoCHA) study (1 April 2014 to 31 March 2017) will address these deficiencies in the evidence base. Its objectives are:

1. To measure the relative performance (cost efficiency) of rehabilitation services in community hospitals;
2. To identify the characteristics of community hospital inpatient rehabilitation for older people that optimise performance;
3. To investigate the current impact of community hospital inpatient care for older people on secondary care and the potential impact if community hospital rehabilitation was optimised to best practice nationally;
4. To examine the relationship between the configuration of intermediate care and secondary care bed use;
5. To develop toolkits for commissioners and community hospital providers to optimise performance.

## Methods and analysis

A mixed-methods approach will be taken, combining established quantitative and qualitative techniques, consisting of four interlinked studies. This approach employs the use of existing data alongside complementary prospective data to produce insights that could not be achieved by the study of the components alone, and represents an efficient and effective research design.

*Study 1*: an analysis of cost efficiency among community hospitals.

*Study 2*: a national survey of community hospitals.

*Study 3*: a multimethod comparative case study of purposively selected community hospital wards providing rehabilitation to older people.

*Study 4*: the development of web-based quality improvement tools for community hospitals and commissioners.

**Figure 1:**
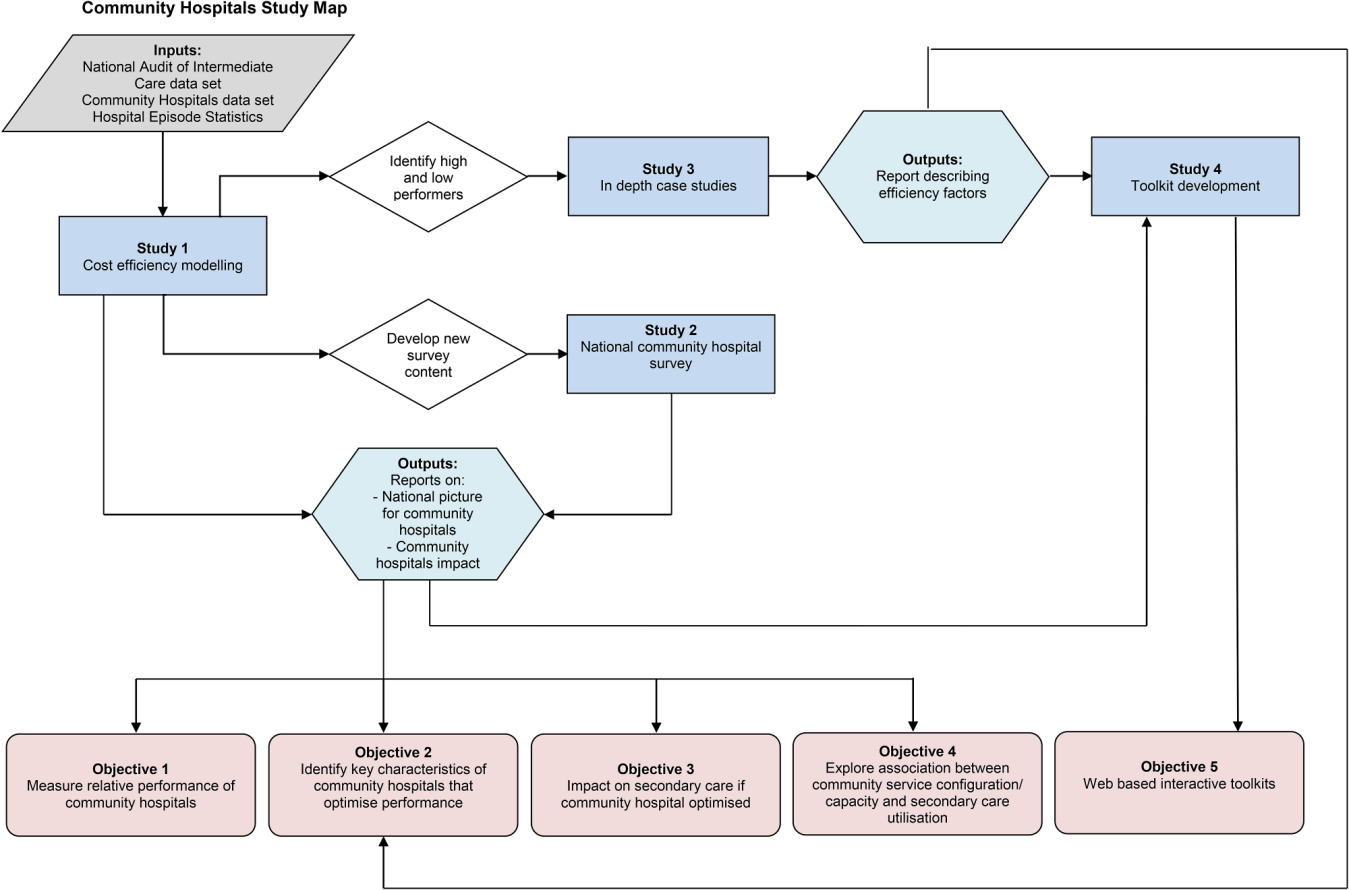
The community hospital study map. HES, Hospital Episode Statistics; NAIC, National Audit of Intermediate Care.

Figure 1, the community hospital study map, illustrates the relationship of the four studies to the five research objectives.

### Study 1: an analysis of cost efficiency among community hospitals

Objective1—to measure the relative performance (cost efficiency) of rehabilitation services in community hospitals.

**Figure 2:**
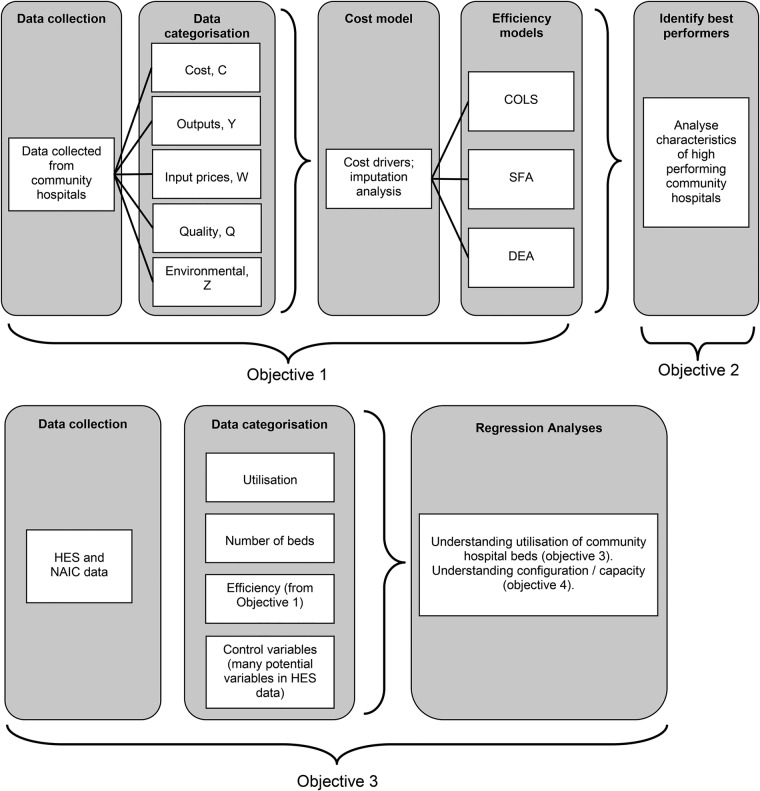
The components of the cost efficiency study (study 1). COLS, corrected ordinary least squares; DEA, Data Envelopment Analysis; HES, Hospital Episode Statistics; NAIC, National Audit of Intermediate Care; SFA, Stochastic Frontier Analysis.

Figure 2 illustrates the various components of study1.

Previous economic evidence on community hospitals derives from a cost-effectiveness analysis embedded within a multicentre randomised controlled trial.^9^ The efficiency analysis we will adopt in this study is different in as far as it provides a framework to assess the extent to which resources that have already been allocated to health services are optimally deployed. A service or process is said to be productively efficient if it produces a given output at the least possible cost.^12^ The aim of this analysis is to identify the performance range for community hospital wards and to explain variations in costs for rehabilitation of older people.

In broad terms, the inputs required for the efficiency analysis include costs (operating costs), output (eg, occupied bed days) and quality (eg, the frequency of multidisciplinary team meetings) data, and the outputs are community hospital-specific efficiency scores; an assessment of the scope for efficiency gains across the sector; and information for how costs vary with important variables such as scale and quality. The data sources used in this study are from the NAIC and the Community Hospitals NHSBN data sets, and Hospital Episode Statistics (HES) as described in table 1. Data from the NAIC and NHSBN Community Hospitals are collected directly from commissioner and provider organisations via an online data collection tool. Data are held by the NHSBN in a SQL database. The NHSBN undertakes validation checks to ensure data quality. Data collection is annual. The data will be provided to the research project in CSV files in anonymised format. A subcontract is in place with East London NHS Foundation Trust, the NHSBN's host organisation, for reimbursement to the Network of the costs of the Community Hospitals data collection.

**Table 1:** Data sources

Data	Source(s)	Features	Collection
National Audit of Intermediate Care	British Geriatrics Society; the Association of Directors of Adult social Services; AGILE (chartered physiotherapists working with older people); the Royal College of Physicians; the Royal College of Nursing; the Royal College of Speech and Language Therapists; the Patients Association; and NHSBN	Commissioners (62) and providers (112) in round 1, round 2 has higher participation (92 commissioners, provider numbers need validation); 370 Intermediate care services, both home-based and bed-based; information on demography (age, gender, preadmission accommodation, place of referral), level of required care, clinical outcomes, service outcomes, PREM	Directly from services
Community Hospitals NHSBN data sets	NHSBN	Opt-in scheme for NHSBN members; around 180 community hospitals; 2 years of data; information on workforce, activity, investment levels, organisational features, services provided and quality measures	Survey of community hospitals by NHSBN
HES	HSCIC	Patient-level data of hospital admissions; collected in episodes of care; range of episode-specific and patient-specific information available	Recorded at all secondary care providers

HES, Hospital Episode Statistics; HSCIC, Health and Social Care Information Centre; NHS, National Health Service; NHSBN, NHS Benchmarking Network; PREM, patient-reported experience measure.

Data are available at the whole hospital level which includes the full range of services and at the ward level which is on rehabilitation services only. These services constitute a large proportion (circa 70%) of the total activity of community hospitals. Analysis at this level allows for greater comparability between community hospitals because services are very similar. Therefore, more reliable estimates of efficiency are derived. To support this claim, we have analysed hospital-level data in preliminary modelling, but the results were implausible. This was not the case when the ward-level data were analysed. Ward-level analysis seems more appropriate empirically. Also, it may be of greater use to managers who have a clearer idea as to the source of inefficiency.

We will investigate the variation in efficiency across community hospitals wards using a range of well-established but analytically complex methods including corrected ordinary least squares (COLS) regression analysis, Stochastic Frontier Analysis (SFA) and Data Envelopment Analysis (DEA). COLS regression models enable relationships between (in this case) costs and cost drivers to be estimated, thus revealing the extent to which changing cost drivers will impact on cost. SFA is a similar approach that likewise investigates relationships between costs and the cost drivers. However, in this model, the estimated relationship is interpreted as an efficiency frontier, and community hospital ward inefficiency is measured against that frontier. SFA is widely used in the academic literature^13^ and is also used by some economic regulators in the UK and internationally.^14^ SFA has been used recently in the NHS setting.^15^ ^16^ DEA is a method which uses mathematical-programming techniques to produce an efficiency score for each unit analysed.^17^ These techniques have been extensively applied to the health sector in general and to the hospital sector in particular.^18^

COLS and SFA are described as below.1
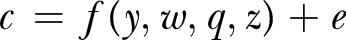
2
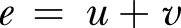


Where,

c—costs: ward-level operating costs; exclusive of capital.

y—Outputs: a measure of the services provided, for example, occupied bed days, admissions, discharges (these are commonly used in health cost analyses^18^). For modelling, we prefer occupied bed days; other measures are used for sensitivity analysis.

w—Input prices: as we have operating costs, we have input prices for labour and materials.

q—Quality: measures to capture the quality of services, for example, physio/OT ratio (as recommended by our clinicians; a unique measure for community hospitals), frequency of staff meetings, whether the community hospital is engaged in research. We also apply measures to capture unobserved heterogeneity for any quality that is not captured by these variables or is imperfectly measured.

z—Observable heterogeneity: differences between services for which we have data, including case mix, risk factors and hospital characteristics, for example, service variables (as a proxy for case mix), admission criteria for the community hospital, length of stay (a proxy for risk factors), the age of the community hospital, inter alia. We also apply measures to capture unobserved heterogeneity for any differences between services that are not captured by these variables or are imperfectly measured.

The f(…) represents the cost function. This is the relationship between costs and drivers of costs. That is, the factors in the brackets in equation (1) explain differences in costs between community hospitals; these factors are removed from our measure of inefficiency.

e—Error term or model residual—the remainder of costs that are not explained by the cost function. In the COLS model, this is used to compute the inefficiency.

u—Inefficiency in the stochastic frontier model (the SFA model is an extension of COLS that allows the separation of inefficiency from random noise^19^).

v—Random statistical noise (eg, measurement error, untoward events).

It is important to deal with variations in costs for which data are not available (or variables that are imperfectly measured; eg, service quality, which is difficult to define and to measure). This is termed unobserved heterogeneity in economic jargon. It is possible to use econometric methods to do this in four broad ways, which have been used in healthcare settings:^15^ ^16^

1. The stochastic frontier model can accommodate random noise, which, in part, comprises unobserved heterogeneity;
2. Using panel data, allowing for community hospital-specific effects to control for unobserved heterogeneity;
3. The use of the Mundlak approach to dealing with unobserved heterogeneity;
4. The latent class stochastic frontier model.

The specific approach is an empirical issue. Statistical testing is used for model specification.

In sum, econometric methods allow us to: measure the relationship between costs and cost drivers (eg, output), including measuring economies of scale; control for observed and unobserved differences between community hospitals; separate out random noise from efficiency estimates; and estimate the relative cost efficiency of rehabilitation services for older people provided in the community hospital.

We anticipate some missing data and propose to address this as follows. We first categorise our variables into groups according to economic theory: costs, outputs, input prices, environmental variables and quality.^13^ We then make an assessment of the extent of missing data and decide on variables to be used based on initial statistical testing and consultation with the research group members. Next, we conduct modelling based on the base data set (ie, unmodified data) and on an imputed data set as modified by a range of standard imputation methods.^20^ We conduct appropriate sensitivity analysis and select our preferred model. We therefore set out the following procedure for dealing with missing data items:

1. Categorise variables according to economic taxonomy;
2. Analyse and summarise missing data;
3. Decide on final set of variables for modelling;
4. Run a range of models on base data (no imputation);
5. Run a range of models on imputed data;
6. Conduct statistical testing/sensitivity analysis;
7. Selection of preferred model.

Objective 2—to identify the characteristics of community hospital inpatient rehabilitation for older people that optimise performance.

The analyses described for objective 1 identify, after taking account of differences between hospitals as captured by the range of cost drivers, the community hospitals that perform best and provide an estimate for the gap between the higher performing community hospital wards and the others. Thus, ‘best’ care in this health economic context is defined by describing the features of those hospitals that have the highest relative efficiency.

Objective 3—to investigate the current impact of community hospital inpatient care for older people on secondary care and the potential impact if community hospital rehabilitation was optimised to best practice nationally.

A multiple regression analysis will be used to investigate the relationship between secondary care usage, identified in the HES data, and relevant variables characterising the quantum and nature of community hospital care within the relevant Clinical Commissioning Group (CCG) area. We use the standard regression approach rather than frontier-based approaches as the focus is on how the explanatory variables impact on the dependent variable, secondary care usage, rather than on the efficiency of certain providers. The community hospital variables of interest are the number of community hospital beds used for the rehabilitation of older people and parameters of efficiency and quality of care as identified in study 1.

Objective 4—to examine the relationship between the configuration of intermediate care and secondary care bed use.

We will adopt a whole system perspective in order to determine if there is an association between the configuration of intermediate care (community-based rehabilitation) services and secondary care usage by older people. This analysis will use the NAIC and HES data sets. A standard regression model will be used to estimate the relationship between secondary care usage (sourced from HES) and key variables sourced from NAIC capturing the configuration of community-based rehabilitation services (the capacity of intermediate care: community hospitals, home-based rehabilitation, care home rehabilitation and reablement services). The way in which community-based rehabilitation services interact with each other, and with the wider secondary care system, is complex and a key part of the project will be to determine how to capture this complexity into a set of measures for inclusion in the model.

### Study 2: national survey of community hospitals

Objective 3—to investigate the current impact of community hospital inpatient care for older people on secondary care and the potential impact if the community hospital rehabilitation was optimised to best practice nationally.

Two existing data sets for this research programme, the NAIC and the NHSBN Community Hospital data sets, comprise only 2/3 of UK community hospitals. The purpose of this national survey of community hospitals is to draw on the findings of study 1 and to use a brief survey instrument to obtain a complete description UK community hospital rehabilitation practice. The survey will focus on those variables most strongly associated with community hospital efficiency (eg, total occupied bed days, input prices, staff mix and bed occupancy), allowing inferences to be drawn about the scale of the work to optimise community hospital ward care nationally. The results will help CCGs to plan strategic changes to community rehabilitation services. The survey instrument will be posted with telephone reminders and will use existing or modified questions from the previous NHSBN Community Hospital surveys that align to the key performance features identified in the efficiency models in study 1.

Participants to the survey will be recruited by the NHSBN through advertising the project to its 337 Network member organisations and an inventory of UK community hospitals recently updated by the Birmingham Community Hospital Study Group and the Community Hospitals Association. Ward staff supported by the audit departments of participating organisations will complete the survey, online—as is usual practice in NHSBN surveys, completing one survey form for each rehabilitation ward in the hospital. A second round using a shorter survey instrument including only the variables required to calculate the cost efficiency model will be directed at the non-responders.

The preferred performance (cost efficiency) model will be applied to the results of study 2 which, being more up to date and complete than the historical data used to produce the models, will enable the most complete and accurate estimate of the performance of community hospitals and enable the best estimate of the likely consequence across the country of optimising the performance of community hospitals (objective 3).

### Study 3: in-depth case studies

Objective 2—to identify the characteristics of community hospital rehabilitation that optimise performance.

Using a comparative case study design,^21–23^ we will draw on multiple methods and perspectives better to understand current inpatient community hospital care and contribute to an understanding of what best cost-efficient performance looks like in practice. This method was previously employed successfully in a study of intermediate care.^24^

Drawing on study 1, we will purposively select three case studies of community hospital wards providing rehabilitation to older people. We will identify two high performers and one low performer based on relative efficiency from study 1, to enable in-depth exploration of how cases that differ in respect of performance vary in terms of care delivery and user-centred outcomes.

Within case studies, we will employ maximum variation sampling^25^ of staff to ensure inclusion of staff members from different disciplines and seniority levels. We will also purposively select a sample of older patients and their caregiver (5/6 patient/caregiver dyads in each case study), and follow them from admission to discharge via observation, conversations/interviews and medical records. Selection will be based on typical and critical case sampling strategies—patients that are typical of those on community hospital wards and critical in that they pose particularly difficult challenges for delivery, such as cognitive impairment.

We will employ multiple quantitative and qualitative data collection methods to build up a picture of each community hospital case, the design and execution of which will incorporate processes for ensuring rigour and quality.^26^ This will include ward patient profiles from routinely collected aggregate data and an assessment of care culture (shared philosophy, leadership, mutual support and team working) using a structured questionnaire with staff.^27^ We will observe practice, including the work of therapy, nursing and medical staff, interactions between professionals and between professionals and patients, decision-making and discharge planning (30–40 hours in each case study). Detailed descriptions of settings, events, interactions and activities will be maintained in field notes. Emerging categories about the data will be tested through more focused observation.

Qualitative interviews with staff (6–7 in each ward) will include how the work of rehabilitation and care for older people is understood, what makes it work and for whom, the resources available and the professional, organisational, cultural and other contextual factors affecting delivery from their different perspectives. Through discussion of anonymised cases we will explore the kinds of patients perceived as best suited to the service and those most likely to benefit.

Data will be collected about the experience of care from a patient and caregiver perspective via observation of care delivery on the ward and of multidisciplinary team and family meetings and contemporaneous conversations with patients. This approach is particularly valuable, for example, for patients with cognitive problems or dementia, since data are collected in real time it does not require either verbal facility or ability to recall.

We will interview patients selected for observation and their caregivers, in each case study site shortly before discharge from the community hospital to reduce problems of recall. As data analysis will proceed simultaneously with data collection, we will leave open the possibility of undertaking a small number of additional interviews to pursue promising lines of enquiry not anticipated in advance. The interviews will assess if and how the care they received facilitated recovery from their perspective.

### Data analysis will be in six stages

*Stage 1*: With the ward as the unit of analysis, we will construct a narrative description of the structure (bed base, staffing, patient profile), activities (throughput) and care culture. Quantitative data from the hospital admission systems (age, sex, reason for admission, type of residence, length of inpatient stay, discharge destination, hospital mortality) will be analysed to provide descriptive statistics, while qualitative staff interview data and observational data will be drawn on to examine how beliefs and values are translated into practice in each organisation.

*Stage 2*: Employing the grounded theory analytical techniques,^28^ such as simultaneous data collection and analysis, constant comparison and search for negative cases, we will examine the process of delivery of rehabilitation care to patients with different characteristics and needs in the real-life context of the ward environment by drawing on the observational data and conversations with staff and patients. Such grounded theory techniques provide a robust approach to analysis and direct attention on conditions, processes and consequences pertinent to this study.

Stage 3: With the patient as the unit of analysis, we will similarly employ the grounded theory analytical techniques to compare and contrast experiences and outcomes across patients similar to, and different from, each other in terms of the nature of the event that precipitated admission and their prior characteristics (such as cognitive impairment). We will refer to the recovery trajectories developed in research on intermediate care^29^ which take account of the diversity of patient characteristics and what recovery means from the perspective of the patient.

Stage 4: Using analytic induction, we will compare and contrast cases in their structure, culture and process of delivery drawing on the narrative descriptions of each case from stages 1 and 2. We will specifically focus on features of structure, culture and delivery processes that differentiate between high-performing and low-performing cases.

*Stage 5*: Involves synthesising and simplifying the conditions and delivery processes from stage 4 to begin to explore the relationship between those conditions, delivery processes and patient outcomes from stage 3. This will involve the use of Qualitative Comparative Analysis (QCA) techniques^30^ ^31^ to identify which key factors are logically necessary and sufficient to result in these recovery trajectories or outcomes.

*Stage 6*: We will review the different causal paths resulting from the QCA analysis and then test them out through further perusal of the case narratives. The outcomes of the case studies will be examined alongside the cost efficiency analysis to explore and seek to account for, discrepancies in the qualitative and quantitative studies. The final part of the analysis will focus on locating the community hospital rehabilitation within the broader intermediate care system for older people.

### Study 4—to develop toolkits for commissioners and community hospital providers to optimise performance (objective 5)

We will develop two web-based interactive toolkits for use by local commissioners and community hospital teams, respectively, that support operational changes to optimise their community hospital wards. The web pages for the online toolkits will be built in asp.net and linked to the Network's SQL Server database which contains the NAIC and the NHSBN Community Hospital Programme data. The toolkits will be securely accessible via the NHSBN website. The toolkits will be based on previous similar work conducted by NHSBN for other clinical service areas and will include three core elements: local performance indicators benchmarked against best performance; a calculation of local potential performance gains and case studies.

The key indicators of community hospitals that optimise performance will be presented in a series of dashboards. The dashboards will be customised to suit the different needs of commissioners and providers. The dashboards will show the national performance range, the performance level of the best performing community hospitals and local performance against each key indicator. The dashboards will show summary information for all community hospitals in the particular health economy with the ability to drill down to the performance of individual community hospitals and additional performance metrics.

A Quality, Innovation, Productivity, and Prevention (QIPP) calculator will show the gap between local and best performing community hospitals, the investment required to meet best practice levels of community hospital capacity and potential savings from meeting best practice efficiency values. The potential impact on local secondary care of optimising community hospital capacity and performance will be modelled.

Descriptions of the in-depth case studies generated in study 3 will be available for download as part of the toolkit. The case studies will explain how the best performing community hospitals achieved high levels of performance.

The content of the toolkits will be co-produced and iteratively modified with a group of 3–6 community hospital teams, the Community Hospitals Association and the Patients Association. The toolkits will be launched at workshops within the final conference event and delegate suggestions for further modification considered.

Initial testing will be conducted with the group of 3–6 community hospital teams by the NHSBN analytics team. The web pages will then be published to the testing area of the Network's servers. A further round of testing will take place with 3–6 new community hospital sites (commissioners and providers). Amendments can be made at this stage to ensure the toolkits function as specified and meet user requirements.

Once testing is complete, the toolkit will be published to Network's live web and database environments.

## Ethics and dissemination

Study 3 required ethical approval as patient consent is necessary to obtain information from medical records about individual outcomes, to undertake specific ward observations relating to their care, and to carry out qualitative interviews/conversations with them and their caregivers about their experiences of care in the ward. Ethical approval was obtained via the Health Research Authority that governs research ethics in the UK (Reference: 15/YH/0062). The main ethical issue is one of consent to participate among patients who may lack capacity on account of dementia or delirium. To exclude such patients from the study risks the loss of voices of those for whom care delivery may be particularly problematic. The consent procedures will adhere to the Mental Capacity Act (2005) and accompanying Code of Practice. First, where an individual has been identified by staff as having a condition that could affect their mental capacity, we will consider how best to approach the person, with advice from ward staff (eg, times when the person may be more alert. or when their relative is present). Second, the researcher will explain the study in clear terms and ascertain whether the person understands the information. Third, if the person is deemed unable to make a decision about participation in the research, we will identify a suitable personal consultee either with the patient or with a staff member (close relative or friend). If there is no suitable personal consultee, a nominated consultee will be consulted to enable patients without next of kin to participate. If the consultee advises that a patient who lacks capacity would be willing to take part in the study then that person will be included in the research, providing that they show no signs of unwillingness to participate (eg, becoming distressed, upset or anxious in the presence of the researcher or when discussing the study). Consent will be an ongoing process. The researcher will repeatedly check with participants that they are happy to continue and will be sensitive to any signs of distress or unwillingness to proceed. If any such signs verbal or non-verbal are present, we will discontinue.

The academic outputs of the overall study will be published both as a report to the funder and in peer-reviewed papers. Dissemination of these and associated reports and other outputs such as the toolkits will be disseminated through our stakeholder networks including the NHS Benchmarking Agency and the Community Hospitals Association.
